# Predictive Potentials of ZEB1-AS1 in Colorectal Cancer Prognosis and Their Correlation with Immunotherapy

**DOI:** 10.1155/2022/1084555

**Published:** 2022-06-25

**Authors:** Liqin Ruan, Weili Chen, Xinhua Zhao, Ningbo Fang, Taiyuan Li

**Affiliations:** ^1^The First Affiliated Hospital of Nanchang University, Nanchang, Jiangxi 330000, China; ^2^Jiujiang First People's Hospital, Jiujiang, Jiangxi 332000, China

## Abstract

**Background:**

CRC is the third most common cancer globally. The tumor immune microenvironment is closely associated with the overexpressed lncRNA ZEB1-AS1. However, in individuals with CRC, the ZEB1-AS1 gene's ability to predict immune response is a mystery.

**Materials and Methods:**

The ZEB1-AS1 gene's prognostic potential was thoroughly investigated. We analyzed and included into the TCGA database all ZEB1-AS1 and ZEB1-AS1-related genes using LASSO-Cox regression. Researchers examined the link among ZEB1-AS1 and the tumor immune microenvironment, immune checkpoint, and tumor mutation burden (TMB) in CRC through the TCGA database. Using a predictive model, researchers were able to determine the link between ZEB1-AS1 and NUDT3 and CRC prognosis.

**Result:**

According to our findings, individuals with reduced ZEB1-AS1 expression had a better prognosis in CRC. Based on the expression of two genes in the TCGA database, patients were divided into two cohorts. The B lymphocytes and macrophages are less likely to be recruited by tissues with a low-risk score. TMB and immunological checkpoints were shown to have a connection. Based on these genes, a predictive nomogram was built and confirmed, with a C-index of 0.78.

**Conclusion:**

Prognostic models based on ZEB1-AS1 and ZEB1-AS1-related genes are more accurate for CRC patients when it comes to the prognosis and immune checkpoint responsiveness.

## 1. Introduction

Colorectal cancer (CRC), one of the usual malignancies, has a high death rate and a younger average beginning age than any other malignancy in China [1]. More than 53,000 people will die from colon cancer in the United States in 2020, according to Cancer Statistics [2]. However, 90% of patients with stage I CRC had a 90 percent five-year relative survival, and just 14% of those with stage IV CRC had a 90 percent five-year relative survival [2]. Advances in targeted and immunotherapy have prolonged the survival of advanced CRC patients [3, 4]. As a result, we have a tough time diagnosing and treating individuals in the intermediate and severe phases of the disease [5]. CRC is still a mystery as to what causes it and how it progresses.

Biological activities of tumor cells were partly regulated by long noncoding RNAs (lncRNAs) which were longer than 200 nucleotides. lncRNAs also can regard as competing endogenous RNA (ceRNA) to regulate miRNAs by targeting the molecule's downstream genes [6]. According to recent findings, lncRNAs are involved in the development and progression of CRC [7]. In 2015, ZEB1-AS1 was found in human HCC [8]. The upregulation was due to hypomethylation of the ZEB1-AS1 promoter in HCC, especially in metastatic tumor tissues. This group has a dismal prognosis for patients with the ZEB1-AS1 overexpression. ZEB1-AS1 can increase cancer cell activities [8]. Meanwhile, ZEB1-AS1 has since been linked to bladder cancer [9], prostate cancer [10], and gastric cancer [11]. The link between stage-related ZEB1-AS1 and CRC, on the other hand, is seldom recorded.

In the current investigation, we discovered that lncRNA ZEB1-AS1 was elevated in CRC tissues, which was unexpected. Meanwhile, we created a predictive model using the relevant gene from public databases and investigated the possible link among ZEB1-AS1 and tumor microenvironment, immune cell infiltration, immune checkpoints, and functional enrichment. Finally, we explored the relationship between the risk score and the tumor mutation burden (TMB).

## 2. Methods and Materials

### 2.1. Data Acquisition

The information was taken from the TCGA database (https://portal.gdc.cancer) and included sequencing of RNA from 647 CRC specimens and 51 normal tissue samples. More than three-quarters of the patients were placed in training groups, while the others were tested (30%). Additional samples were ruled out because they had insufficient information about their patients. A local ethics committee was not necessary since TCGA databases are open to the public and because this study complied with all applicable database access requirements and publication limitations to the letter of the law.

### 2.2. The Expression of ZEB1-AS1 and Its Related Clinical Parameters and Pathways

In order to draw the Kaplan–Meier curves comparing the TCGA cohort groups, the “survminer” R tool was utilized. The “limma” R software identified the amounts of limma that were expressed differentially in various groups. Thermography using the R package “ComplexHeatmap” was accustomed to illustrate the correlation between clinical parameters. To examine whether ZEB1-AS1 was an independent predictor of overall survival, univariate and multivariate Cox regression models were used. By using the clusterProfiler tool, the researchers were able to investigate the ZEB1-AS1 pathway using gene set enrichment analysis (GSEA).

### 2.3. Prognosis Screening and Identification of a Signature Related to CRC Prognosis

Public databases (https://starbase.sysu.edu.cn/and http://m6a2target.canceromics.org/#/) provided the genes targeted upstream and downstream. A coexpression study was also carried out to discover the association between ZEB1-AS1 and relevant genes, which was plotted using Cytoscape v3.8.2 to uncover the regulatory network of ZEB1-AS1. The regulatory network genes that vary between CRC tissues and surrounding nontumorous tissues have been discovered. The overall survival (OS) data were analyzed using a univariate Cox model.

The “glmnet” R package was used in the training group to add ZEB1-AS1 and overlapping prognostic different expression genes into the LASSO-Cox model. The penalty parameter (*λ*) was derived using tenfold cross-validation in accordance with the bare minimal requirements. Gene expression and regression coefficients were used to establish each patient's risk score: Risk score = *e*^sum (each gene's expression × corresponding coefficient)^. We divide CRC patients into two groups by the median risk score of each patient. “t-SNE” R program was used to examine both the groups' distribution.

### 2.4. Building and Testing the Predictive Nomogram

Compared to other clinical factors from TCGA, we tested the risk score as an independent predictor of overall survival using Cox regression (OS). The “rms” R software created a nomogram and calibration maps under independent prediction criteria. The “timeROC” R program was used to conduct ROC curve analysis to determine the nomogram's predictive capacity over time. The testing cohort's patients were examined using the same formula in the training cohort. Analyses were performed on the predictive nomogram to assess the accuracy and specificity of it.

### 2.5. Predictions of the Immunotherapy Response Using Functional Enrichment Analysis

The researchers used ssGSEA and the “GSVA” R package to examine the infiltration and activation of 16 immune cells and 13 immune-related pathways.

As part of the tumor immune evasion (TIDE) project (http://tide.dfci.harvard.edu/), a computer model of the tumor's ability to evade detection includes a T-cell expression profile. For CRC patients, the TIDE algorithm was utilized to determine the effects of immune checkpoint blockade (ICB).

### 2.6. TMB Value Estimation and Prognostic Analysis

The TCGA cohort's somatic mutation distribution was examined using the maftools software. The risk score and TMB were also examined in relation to the prognosis.

### 2.7. Statistical Analysis

In order to conduct statistical studies, we used the R program (version 4.1.1). Tissues from CRC patients were compared to healthy tissues in a two-tailed *t*-test conducted by the student. The Kaplan–Meier method and the log-rank test were used to compare the overall survival of the different groups. In this study, the ssGSEA scores of immune pathways or cells were analyzed using the Mann–Whitney test. All *P* values were two-tailed in this study. In the absence of any other cutoffs, a *P* value less than 0.05 was considered statistically significant.

## 3. Result

### 3.1. The Expression of ZEB1-AS1 in the Tumor

**Figure 1:**
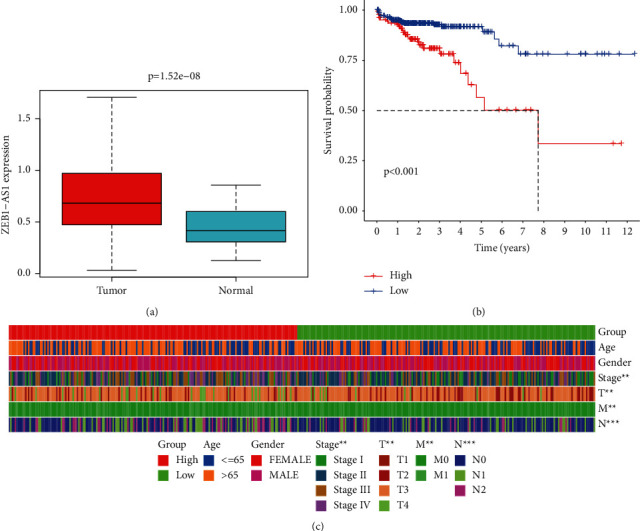
Expression, survival curves, and association of ZEB1-AS1 genes in colorectal cancer (CRC). (a) Comparing ZEB1-AS1 expression across various organs. (b) The Kaplan–Meier curves from the TCGA database demonstrating the OS of patients with high and low ZEB1-AS1 expressions are shown in the image below. (c) Heat map showing the differences in ZEB1-AS1 expression levels between the high and low expression groups. The abscissa represents the sample, whereas the ordinates are clinical features (^*∗*^*P* < 0.05; ^*∗∗*^*P* < 0.01; ^*∗∗∗*^*P* < 0.001; ns: not significant).

We used data from TCGA to examine the clinical characteristics of 647 colorectal cancer patients and 51 normal tissue samples in order to assess the ZEB1-AS1 expression. In malignancies, ZEB1-AS1 is more numerous, and patients with less ZEB1-AS1 have a higher life expectancy (Figures 1(a) and 1(b)). Furthermore, we discovered that a high ZEB1-AS1 expression was linked to advanced T, N, and M status and stages using grouping analysis (Figure 1(c)).

### 3.2. Exploring the Regulated Pathway of ZEB1-AS1

**Figure 2:**
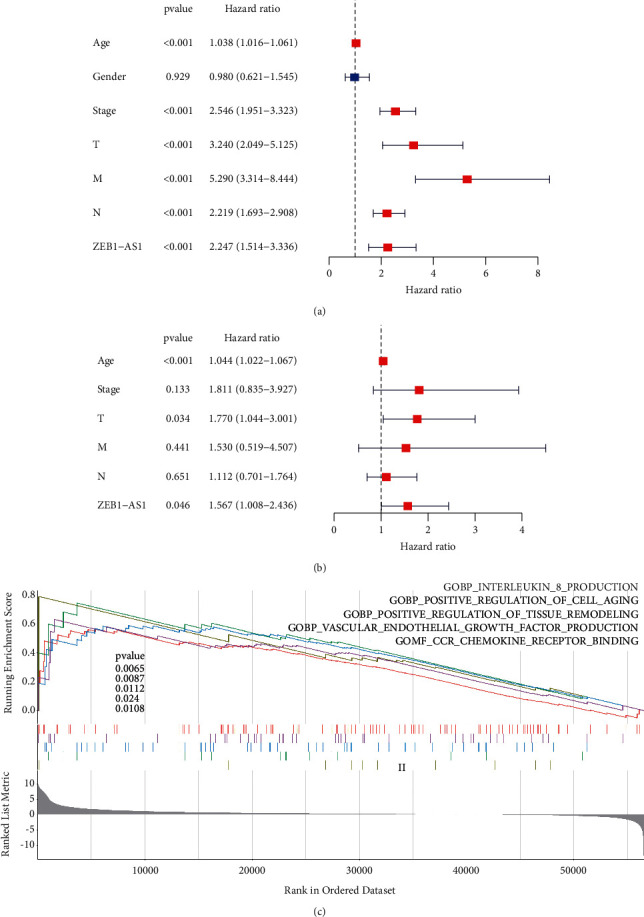
Regulated correlation, univariate, and multivariate Cox analysis of the risk score in CRC. (a) Univariate survival-related analysis was used to discover the prognostic significance of different clinical parameters and ZEB1-AS1 in CRC. (b) Detection of prognostic significance of different clinical variables and ZEB1-AS1 in CRC by multivariate survival-related analysis. (c) The ZEB1-AS1 regulatory pathway.

Univariate and multivariate Cox regression analyses suggested that the lncRNA ZEB1-AS1 may be regarded as a prognosis factor in the treatment of CRC. (*P* < 0.05) (Figures 2(a) and 2(b)). To investigate ZEB1-AS1's function, we discovered that ZEB1-AS1 was associated with interleukin-8 production, positive regulation of cell aging, positive regulation of tissue remodeling, vascular endothelial growth factor production, and chemokine receptor (CCR) binding through GESA (Figure 2(c)).

### 3.3. The Related Target Gene of ZEB-AS1

**Figure 3:**
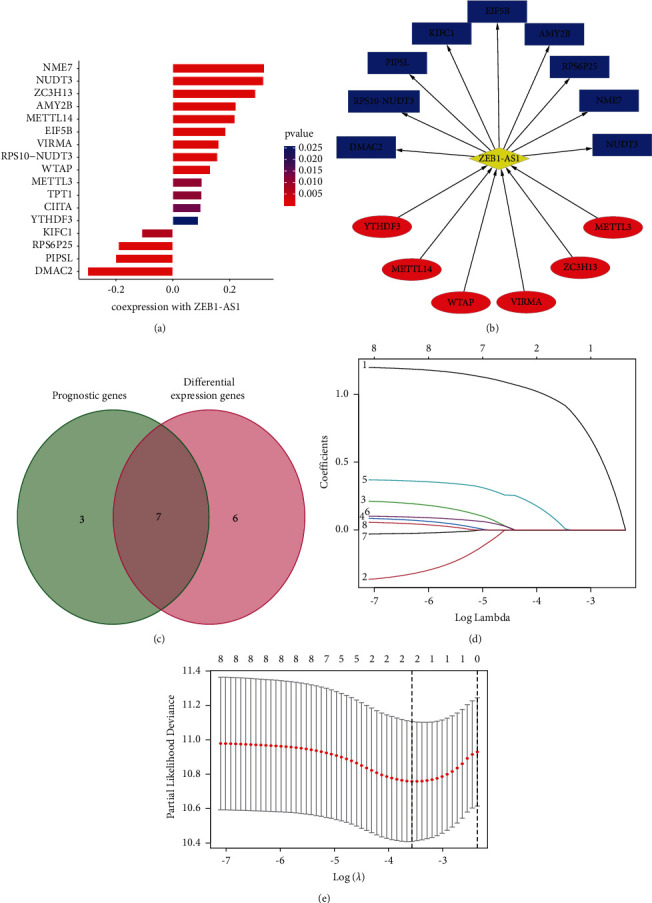
Risk model in CRC patients established according to the ZEB1-AS1 and its related genes in CRC. (a) The various genes that coexpress with ZEB1-AS1. (b) The pattern of ZEB1-AS1 and its regulated genes. (c) Gene-prognostic model interaction in CRC. (d) The error curve could be cross-validated and the tuning parameters (log *λ*) of OS-related proteins were chosen. With the minimum and 1-SE criterion, imaginary lines were drawn at the ideal value that were perpendicular to each other. (e) 8 OS-related genes' LASSO profile was drawn along with a reversing imaginary line at the value found by 2-fold cross-validation.

Our search for a putative ZEB1-AS1 acting site began with public databases (Starbase and M6A2Target). We uncovered the regulated genes of ZEB1-AS1 (Figure 3(a)) and showed their interaction through Cytoscape (Figure 3(b)). Using these genes (METTL14, VIRMA, EIF5B, NUDT3, KIFC1, TPT1, and CIITA), we next looked for variations and prognostic indicators in the seven candidates (Figure 3(c)). The LASSO-Cox regression was used on 7 genes to provide a complete and effective prognosis-risk profile signification. ZEB1-AS1 and NUDT3 were two genes that stood out (Figures 3(d) and 3(e)).

### 3.4. The Risk Scores Were Calculated Based on Seven Genes and ZEB1-AS1

**Figure 4:**
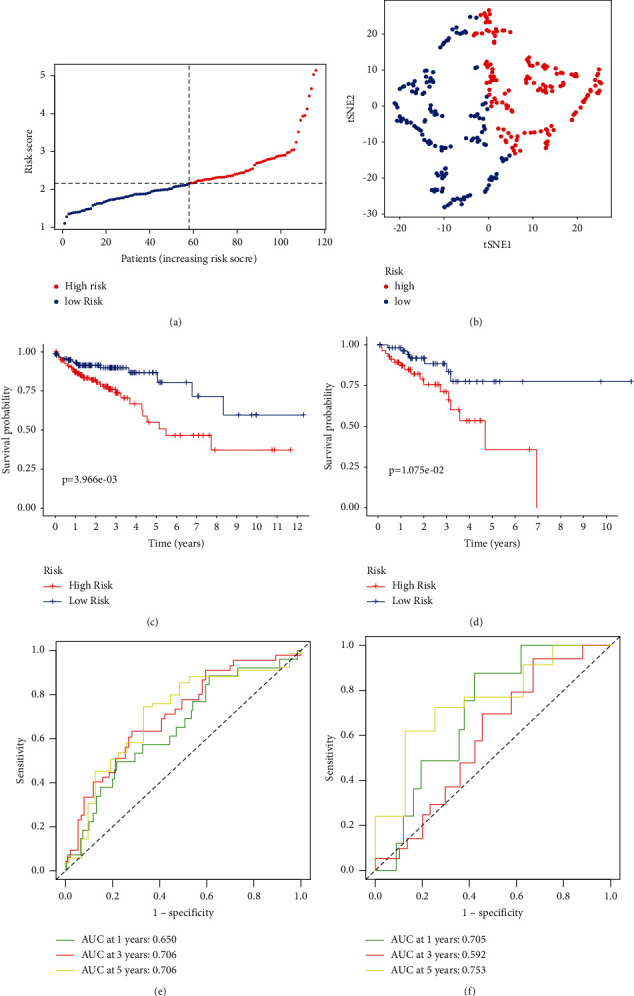
The outcome was predicted by looking at the 2-gene risk level in the TCGA database. (a) The number of risk scores in the TCGA database and the middle score. (b) tSNE analysis of the TCGA database. (c) Kaplan–Meier graphs for overall survival of high-risk and low-risk patients in the training cohort. (d) In the testing cohort, the Kaplan–Meier curves for the OS of patients in the high-risk and low-risk groups. (e) ZEB1-AS1 and NUDT3 ROC curves for predicting 1/3/5-year survival in the training cohort. (f) ZEB1-AS1 and NUDT3 ROC curves for predicting 1/3/5-year survival in the testing cohort.

NUDT3 and ZEB1-AS1 were utilized to construct a risk rating system for the CRC prognosis according to the expression patterns of these genes in various groups of patients. This is how we arrived at our risk score: Risk score = *e*^((0.940 × ZEB1−AS1) + (0.046 × NUDT3))^. The median cutoff value to categorize patients into low-risk and high-risk cohorts (Figure 4(a)). According to the results of the t-SNE study, patients were evenly distributed throughout the various groups (Figure 4(b)). There was a substantial difference in comparing the prognosis of low risk individuals to high-risk ones (Figure 4(c), *P* < 0.001), and the similar results were shown in the testing group (Figure 4(d), *P* < 0.001), showing that the signature of the prognostic is very sensitive and specific when it comes to predicting OS. NUDT3 and ZEB1-AS1 were also shown to have a substantial impact on the prognosis of CRC patients. The prognostic significance of ZEB1-AS1 and NUDT3 in CRC patients from the TCGA dataset was confirmed by ROC analysis (training set: 1-year AUC = 0.650, 3-year AUC = 0.706, 5-year AUC = 0.706, Figure 4(e); testing set: 1-year AUC = 0.705, 3-year AUC = 0.592, 5-year AUC = 0.753, Figure 4(f)).

### 3.5. Construction and Validation of the Predictive Nomogram

**Figure 5:**
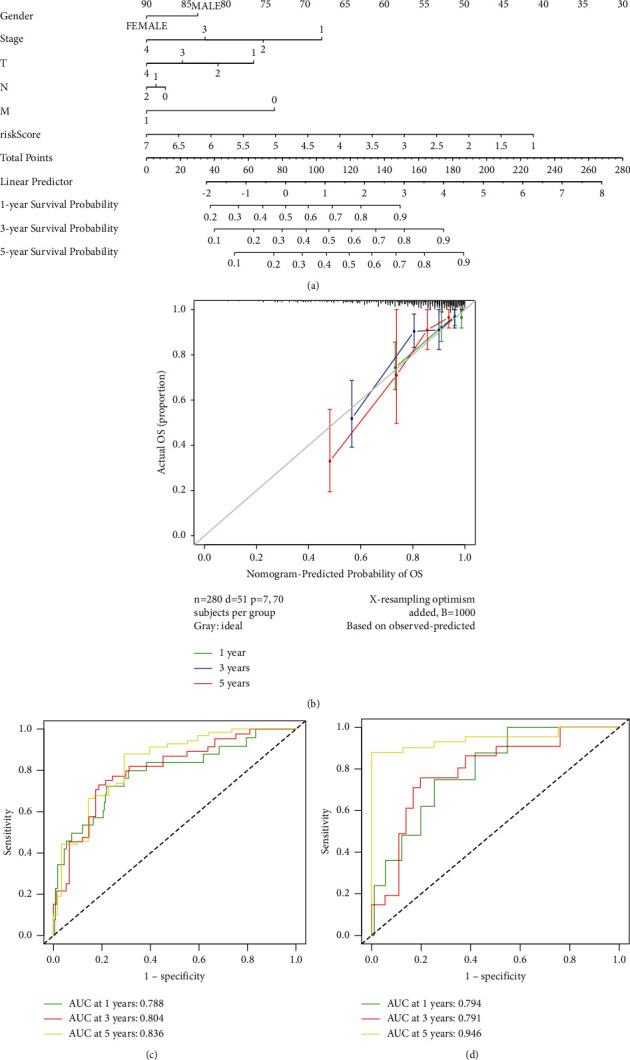
Building and validating a predictive nomogram. (a) A nomogram for predicting overall survival (OS) in colorectal cancer patients at 1, 3, and 5 years. (b) Nomogram calibration curves for OS prediction at 1, 3, and 5 years. (c) ROC analysis with the TCGA training cohort. (d) ROC analysis with the TCGA testing cohort.

In addition, a nomogram based on these independent prognostic indicators was built to estimate individual survival probabilities for 1, 3, and 5 years based on the results of this study (Figure 5(a)). As far as C-index goes, it was 0.78 (95% CI: 0.71–0.85). Calibrating curves demonstrated a strong correlation between predicted OS and observed OS at 1, 3, and 5 years (Figure 5(b)). After that, ROC curves were constructed to confirm the nomogram's ability to predict outcomes. The training cohort's OS AUCs were 0.788, 0.804, and 0.836 (Figure 5(c)). We integrated patients from the testing cohort into the prediction model in order to investigate the model's robustness. Each of the nomogram's AUCs was 0.794, 0.791, and 0.946 (Figure 5(d)).

### 3.6. Comparison of Patients with Different Risk Factors' Immune Infiltration and Immunity

**Figure 6:**
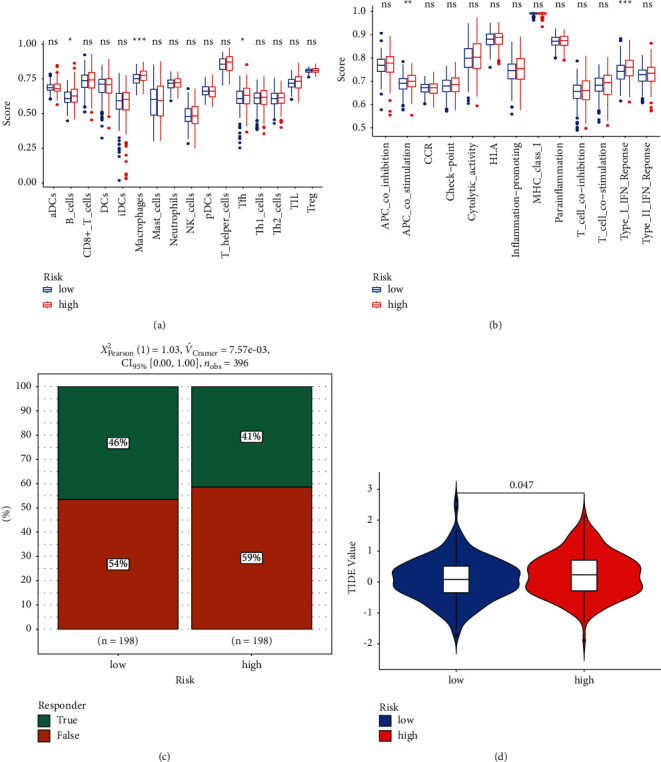
Relationship among the risk score, immune infiltration, immunotherapy response to CRC patients, and tumor immune dysfunction exclusion (TIDE). (a) The scores of 16 immune cells. (b) The scores of 13 immune-related functions. (c) The response to immunotherapy. (d) The value of TIDE (^∗^*P* < 0.05; ^∗∗^*P* < 0.01; ^∗∗∗^*P* < 0.001; ns: not significant).

Several immune cell subsets, activities, and routes were studied to establish the risk score-immunological state link. When comparing high-risk people to those in the low-risk group, B cells, macrophages, and Tfh cells scored substantially higher in the high-risk group (Figure 6(a)). The TCGA database showed that APC co-stimulation and type I interferon response varied across the two groups (Figure 6(b)). We noticed that patients with a high-risk score had a lower response to immunotherapy than those with a low-risk score, which helped us understand the purpose of the risk score in immunotherapy (41% versus 46%) (Figure 6(c)). Meanwhile, the TIDE value also suggested the high-risk score group gets a better immunotherapy response (Figure 6(d)).

### 3.7. The Risk Score Is Associated with Tumor Mutation Burden (TMB) and an Immune Checkpoint

**Figure 7:**
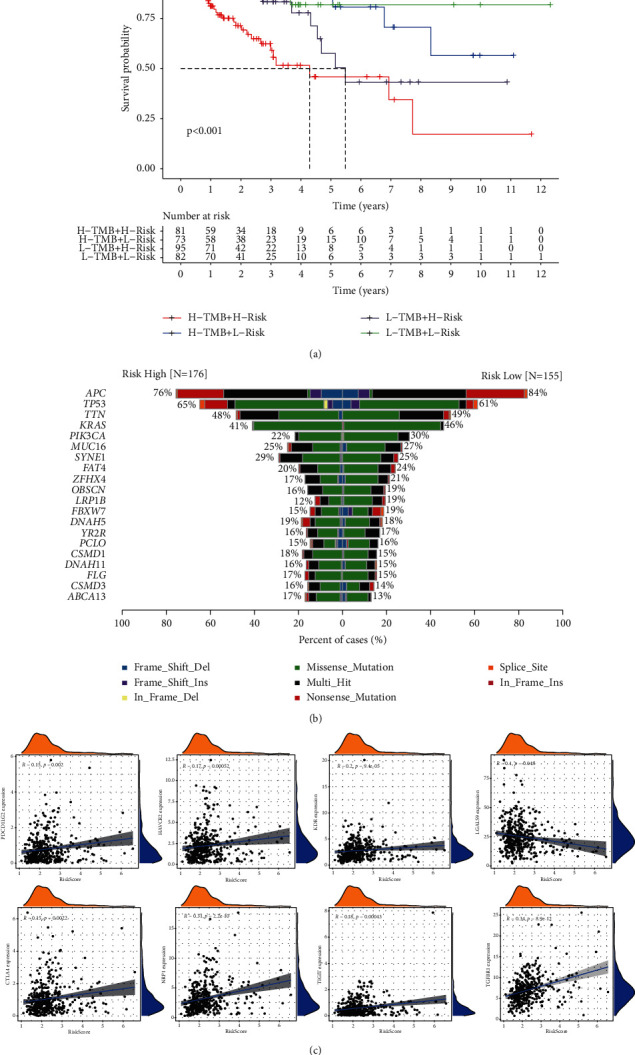
Prognostic risk score correlation with immunity characterization of CRC. (a) The Kaplan–Meier curves for overall patient survival. (b) The TMB in different risk groups. (c) Relationship between the risk score and different immune checkpoints (^*∗*^*P* < 0.05; ^*∗∗*^*P* < 0.01; ^*∗∗∗*^*P* < 0.001; ns: not significant).

To learn more about ZEB1-AS1 and NUDT3, we explored the TMB and immune checkpoint. Our results showed some important genes (APC, KRAS, PIK3CA, etc.) concerning the progress of CRC get more mutations in the low-risk group (Figure 7(b)). Moreover, ZEB1-AS1 and NUDT3 mostly have a positive correlation with major inhibitory immune checkpoints (NRP1, CTLA4, TIGHT, KDR, etc.) (Figure 7(c)). However, in survival analysis, TMB has the greatest survival rate compared to the other groups in a high-risk score (Figure 7(a)). Combining the foregoing findings, it has been hypothesized that those patients that have a high-risk score may have had an aberrant immunological state.

## 4. Discussion

In this study, based on publicly accessible information, the expression of ZEB1-AS1 in CRC tissues was investigated, as well as the association between ZEB1-AS1 expression and overall survival (OS) in CRC patients. NUDT3, a gene linked to ZEB1-AS1, was shown to be associated with a worse outcome in CRC. Based on ZEB1-AS1 and NUDT3, we developed a predictive nomogram that was very accurate in predicting overall survival. In the end, patients with varied risk ratings demonstrated a wide range of immunological and functional enrichment levels. ZEB1-AS1 and NUDT3 were shown to be immune and prognostic biomarkers in CRC, respectively, in our research. Moreover, it is the first NUDT3 and ZEB1-AS1 prediction model for CRC patients.

Different cancers relied heavily on lncRNA. An early lncRNA was found by scientists as H19 (2.7 kb) [12, 13], and hypoxic stress triggers the protein's production, which is then connected to the epithelial-to-mesenchymal transition (EMT), and malignancies, such liver, breast, colorectal, esophageal, lungs, pancreatic, gastric, bladder, and cervical carcinomas, may be triggered by its overexpression, which stimulates angiogenesis, cell survival, and proliferation genes [14, 15]. Aside from controlling the tumor growth, lncRNAs can also influence cytokines. Hepatocellular carcinoma was treated with lncRNA PANDA, which reduced interleukin-8 (IL-8), allowing previously aged cells to continue to multiply and contribute to tumor growth [16]. There was a significant level of expression of ZEB1-AS1 in the tumor and an association with IL-8 in our study, which suggests that IL-8 secretion may be influenced in normal CRC by lncRNA ZEB1-AS1. Cancer patients have greater levels of IL-8 than healthy individuals and the higher IL-8 levels seem to correspond with higher stage, grade, and tumor burden in patients [17, 18]. Thus, our findings demonstrated a connection between ZEB1-AS1 and more advanced stage and grade levels, as expected. In addition, we found that ZEB1-AS1 and NUDT3 were linked to B cell and macrophage infiltration in our research, which may promote tumor immunosuppression. Furthermore, TMB is smaller among those at higher risk, which suggests that immunotherapy was more effective for those at lesser risk [19, 20]. However, the results of immunotherapy response, TIDE, and survival analysis were contrary to TMB and immune infiltration that may be influenced by the gene NUDT3. Moreover, as the biomarkers for tumor immunity, the potential function of NUDT3 and ZEB1-AS1 in tumor immunity is still unclear, which needs further research.

Additionally, we also focus on some genes associated with inhibitory immune checkpoints, like NRP1, CTLA4, and others. Although the correlation coefficient is lower among the risk score and these genes, they regard them as the biomarkers for tumor immunity which is enough combined with our above result. An anti-NRP1 antibody, in combination with an anti-PD-1 antibody, has been shown to improve CD8 T-cell proliferation, cytotoxicity, and tumor control [21]. Antitumor immunity requires the production of long-lasting tumor-specific T cells, which may be boosted by blocking NRP1 [22]. This is consistent with the findings of our prognostic model, which indicates that the survival rate in the high-risk score group is lower than the survival rate in the low-risk score group. Nonetheless, the role of ZEB1-AS1 and its related gene in tumor immunity remains an unanswered question that has to be investigated further.

## 5. Conclusion

In summary, we devised a new predictive model for ZEB1-AS1 and its associated gene in CRC, which has shown a high degree of prognostic relevance for immunotherapy response in patients with CRC. Detailed examination of the mechanisms by which these genes interact with the tumor immune microenvironment in CRC will be required in future research.

## Data Availability

The data used during the current study are available from the corresponding author upon request.
